# Rough-set based learning: Assessing patterns and predictability of anxiety, depression, and sleep scores associated with the use of cannabinoid-based medicine during COVID-19

**DOI:** 10.3389/frai.2023.981953

**Published:** 2023-02-15

**Authors:** Sheela Ramanna, Negin Ashrafi, Evan Loster, Karen Debroni, Shelley Turner

**Affiliations:** ^1^Department of Applied Computer Science, University of Winnipeg, Winnipeg, MB, Canada; ^2^Ekosi Health Centre Corporation, Winnipeg, MB, Canada

**Keywords:** rough sets, machine learning, electronic health records, mental health, cannabinoid medicine, rough-fuzzy sets

## Abstract

Recently, research is emerging highlighting the potential of cannabinoids' beneficial effects related to anxiety, mood, and sleep disorders as well as pointing to an increased use of cannabinoid-based medicines since COVID-19 was declared a pandemic. The objective of this research is 3 fold: i) to evaluate the relationship of the clinical delivery of cannabinoid-based medicine for anxiety, depression and sleep scores by utilizing machine learning specifically rough set methods; ii) to discover patterns based on patient features such as specific cannabinoid recommendations, diagnosis information, decreasing/increasing levels of clinical assessment tools (CAT) scores over a period of time; and iii) to predict whether new patients could potentially experience either an increase or decrease in CAT scores. The dataset for this study was derived from patient visits to Ekosi Health Centres, Canada over a 2 year period including the COVID timeline. Extensive pre-processing and feature engineering was performed. A class feature indicative of their progress or lack thereof due to the treatment received was introduced. Six Rough/Fuzzy-Rough classifiers as well as Random Forest and RIPPER classifiers were trained on the patient dataset using a 10-fold stratified CV method. The highest overall accuracy, sensitivity and specificity measures of over 99% was obtained using the rule-based rough-set learning model. In this study, we have identified rough-set based machine learning model with high accuracy that could be utilized for future studies regarding cannabinoids and precision medicine.

## 1. Introduction

COVID-19 is an unprecedented health crisis causing a great deal of stress and sleep challenges for populations in Canada. Research is emerging highlighting the potential of cannabinoids' beneficial effects related to chronic pain (Lynch and Campbell, 2011), substance use (Hay et al., 2018), addiction (Prud'homme et al., 2015), and poor mental health (Lee et al., 2017; McGuire et al., 2018). Recent studies point to the clinically significant acute impacts the pandemic is having on insomnia rates (Morin and Carrier, 2021). Where there is recent research which points to the potential positive impact cannabinoid may have regarding sleep (Ware and Ferguson, 2015; Sznitman et al., 2020), a 2017 review (Babson et al., 2017) of the literature on cannabinoids and sleep suggested mixed results and highlighted the need for further research.

With the availability of large amounts of patient data, machine learning (ML) techniques, specifically, supervised and deep learning classifiers, have made it possible to detect, diagnose and treat mental health disorders. Common dataset formats include: Electronic health records (EHR) (Ramesh et al., 2021), Social Media (e.g., Twitter, Reddit) (Tariq et al., 2019; Kim et al., 2021), Image (e.g., MRI) (Noor et al., 2020) and Audio (Xiao et al., 2016). Shatte et al. (2019), present an in-depth review of about 300 papers related to ML and its application in mental health. The most common ML models used include: support vector machines, decision trees, naive bayes, k-nearest neighbor, and neural networks (deep learning). Latent Dirichlet allocation (LDA) and sentiment analysis models were used for learning from textual and social media data. Predicting mental health from social media data is an interdisciplinary area also known as *human-centric machine learning* where human insights are combined with data driven predictions (Chancellor et al., 2019a). Ethical tensions in inferring mental health states of individuals from social media data are discussed in Chancellor et al. (2019b). In another study (Edo-Osagie et al., 2020), Twitter data was used in public health in surveillance, detection, and prevention of events. In Sharma and Verbeke (2020) XGboost classifier was used to assess the effectiveness of biomarkers to classify depression cases from healthy cases using a large dataset from Netherlands. In a recent study (Dobias et al., 2022), ML models were used to assess whether the adolescents with depressive symptoms had access to treatments and, if yes, where the treatments were received. Nemesure et al. (2021) proposed an ensemble of six classifiers to predict general anxiety disorder (GAD) and major depressive disorder (MDD) problems. Edgcomb and Zima (2019) discuss application of Natural Language Processing techniques to EHR phenotyping (unstructured text) which contain narrative text such as physician notes for improving mental health services. Rahman et al. (2020) present a survey of papers on mental health detection using ML techniques in Online Social Networks. A review of 2261 articles on the application of deep learning models in mental health outcomes was presented in Su et al. (2020). In this paper, various deep neural network architectures as well as different forms of clinical data (neuroimages, EMR, audio visual and social media) are discussed.

A study by Alghamdi et al. (2018) reported using Gaussian Processes, Support Vector Machines, and Neural Networks algorithm to extract predictive patterns of cannabis use and the onset of first episode psychosis from clinical data. This study does not include any specific medical cannabis product (such as CBD or THC). The cannabis use feature consists of three values (never used, hash, or skunk). Another study by Choi et al. (2021) analyze behavior related to depression and suicide risk using machine learning algorithms (Logistic regression, Random Forest, and K-Nearest Neighbor) in adults that use marijuana (cannabis).

Since 1991, rough set theory has been applied extensively in medical informatics (Pawlak, 1991; hrn, 1999; Pattaraintakorn and Cercone, 2008; Hassanien et al., 2009; Gil Herrera et al., 2015; Pathan et al., 2020). More recently, rough set model was used to analyze outpatient service quality in a hospital setting in China (Du et al., 2021). To the best of our knowledge, there are only a few papers related to the application of rough sets in mental health. In Shusaku and Kudo (2009), rough set theory was used to explore the relationship between human psychological state (scores of a psychological scale) and physiological state (level of the secretory biomarkers). In Nomura et al. (2010), the authors use rough sets instead of conventional linear correlation analysis for mining the relationship between a subjective stress scale and salivary cortisol stress biomarker. In Liu et al. (2011), a hybrid rough set and Taguchi-genetic algorithm (RS-HTGA) was proposed to determine the relationship between mental stress and biomedical signals. The efficacy of their model was tested on a clinical dataset comprising 362 cases (196 male, 166 female). In Liu et al. (2014), the RS-HTGA algorithm achieved sensitivity, specificity, and precision scores of 96%. In Mittal et al. (2014), the authors present an application of rough sets for attribute reduction to identify depressive episodes.

In this research, we seek i) to evaluate the relationship of the clinical delivery of cannabinoid-based medicine for anxiety, depression and sleep scores by utilizing machine learning specifically rough set methods; ii) to discover patterns based on patient features such as specific cannabinoid recommendations [includes medical cannabis products contain varying amounts of cannabidiol (CBD) and tetrahydrocannabinol (THC)], diagnosis information, decreasing/increasing levels of clinical assessment tools: GAD-7 (General Anxiety Disorder-7), PHQ-9 (Patient Health Questionnaire-9), and PSQI (Pittsburgh Sleep Quality Index) (Buysse et al., 1988) scores over a period of time including during the COVID timeline; and iii) to predict whether new patients could potentially experience either an increase or decrease in clinical assessment tool scores (pl. see Supplementary Table 4, Appendix A for scale values for each tool). PHQ-9 and GAD-7 scales are well-established instruments for screening for symptoms of depression and generalized anxiety respectively (Kroenke et al., 2001; Spitzer et al., 2006). The dataset for this study was derived from patient visits to Ekosi Health Centres in Manitoba and Ontario, Canada from January, 2019 to April, 2021. Extensive pre-processing and feature engineering was performed on the dataset. To determine the outcome of a patient's treatment, a class feature (Worse, Better, or No Change) indicative of their progress or lack thereof due to the treatment received was introduced. A two-class experiment (Worse or Better) was also explored. Well-known supervised machine learning classification algorithms: Random Forest (tree-based), RIPPER (rule-based) in addition to rough and fuzzy models were trained on the patient dataset. All experiments were conducted using a 10-fold CV stratified method. Also, prediction of new cases using the rough set-based classifier (LEM2 algorithm) is presented. Our results demonstrate that rough-set based classifier (with LEM2 algorithm) is superior to all other tested models in terms of overall classification accuracy (99.34% for the 3-class experiment), accuracy per class, sensitivity, and specificity values for both the 2-class and the 3-class experiments. A statistical *t*-test reveals that there is a difference between rough-set based classifier and other tested classifiers for the 3-class experiment.

Our results support the findings that the combination of THC and CBD appears to be most beneficial on GAD-7, PHQ-9, and PSQI scores for patients dealing with anxiety, depression, sleep disorders, chronic pain, and arthritis. In this study, we have identified rough-set based machine learning model with high accuracy that could be utilized for future studies regarding cannabinoids and precision medicine. This research points to a novel application of rough and fuzzy rough classification learning to a case study involving cannabinoid medicine and anxiety, depression, and sleep pattern data.

## 2. Preliminaries

In this section, we present a brief review of rough and fuzzy rough set theory concepts that were used in this research. Specifically, we use different forms of fuzzy and rough nearest neighbor classification algorithms.

### 2.1. Rough sets

In classical set theory, we can classify whether elements either belong to a set or not. This is a precise or crisp set where the sets have sharp boundaries. However, when boundaries are *unsharp or vague*, it is difficult to classify elements uniquely to one set. In other words, this will result in a boundary region with elements that cannot be classified precisely. Rough set theory was proposed by Zdzislaw Pawlak in early 80's as a mathematical framework to analyze vague data and ill-defined objects based on an indiscernibility or equivalence relation (Pawlak, 1982; Pawlak and Skowron, 1994). Equivalence relations generate equivalence classes and the notion of indiscernibility is defined relative to a given set of attributes (Pawlak and Skowron, 2007). Due to the lack of knowledge (or uncertainty) that objects might belong to more than one set (or class), two approximation operators (*lower* and *upper*) are introduced in rough set theory to generate precise sets. In supervised classification, the advantage of rough set theory is that no prior or additional data is needed to categorize data into classes (Pawlak, 1991). Supplementary Figure 1 shows the regions that emerge with rough set approximation. The lower approximation consists of the objects that certainly belong to the set (orange region) and upper approximation consists of objects that their membership is not certain (green region). The regions are depicted as squares only for the sake of illustration, but they can be of arbitrary shape. We should note that each granule can contain an arbitrary number of objects or may be empty. The oval denotes the target *X* which, in the case of supervised learning, is either a class or a pattern that needs to be learned.

Let *U* be a finite, non-empty universe of objects and let *R*⊆*U*×*U* denote a binary relation on the universe *U*. *R* is called an *indiscernibility relation* and for rough sets, it has to be an *equivalence relation*. The pair (U,R)=A is an *approximation space* A (Stepaniuk, 1998). Let *X*⊆*U* be a target concept in this universe. Then the task is to create an approximated representation for *X* in *U* with the help of *R*. Let [*x*]_*R*_ denote the indiscernibility class of *x* i.e. *y*∈[*x*]_*R*_ ⇔ (*x, y*)∈*R*. Then, every equivalence class forms a *granule* or *partition* containing objects that are indiscernible for this approximation space A. Therefore, every single item in a granule is considered identical and inseparable. These granules are approximated by the following means:

- **Lower approximation**. Intuitively, these are the objects which *certainly* belong to *X* with respect to A.

$$\begin{array}{l} L_{A}\left(X\right)=\left\{x\in U:{\left[x\right]}_{R}\subseteq X\right\}. \end{array}$$

- **Upper approximation**. Intuitively, these are the objects which *may* belong to *X* with respect to A.

$$\begin{array}{l} U_{A}\left(X\right)=\left\{x\in U:{\left[x\right]}_{R}\cap X\neq \emptyset \right\}. \end{array}$$

These two approximations will also form the following two regions:

- **Boundary region**. These are the objects occurring in the upper approximation but not in lower approximation of *X*.

$$\begin{array}{l} B_{A}\left(X\right)=U_{A}\left(X\right)-L_{A}\left(X\right). \end{array}$$

- **Negative region**. These are the objects that certainly don't belong to *X*.

$$\begin{array}{l} U-U_{A}\left(X\right). \end{array}$$

With this framework, we have two different types of sets: a set *X* is called a *crisp set* if and only if $B_{A}\left(X\right)=\emptyset$. Otherwise, it is called a *rough set*. The pair $\left[U_{A}\left(X\right),L_{A}\left(X\right)\right]$ forms the *rough approximation* for *X* (see Supplementary Section 1 for an illustration and list of symbols and their interpretation used in this paper).

### 2.2. Fuzzy rough sets

Fuzzy set theory was proposed by Zadeh (1997) as an extension of traditional set theory to deal with uncertainty and vagueness. In the context of fuzzy sets, let *X* denote the universe, a fuzzy set *A*∈*X* is characterized by a mapping X → [0, 1] which is also called a membership function. A fuzzy relation R in X which is also a fuzzy set and is characterized by a mapping R: X × *X* → [0, 1] (Zadeh, 1997). Del Cerro and Prade (1986), Nakamura (1988), and Dubois and Prade (1990), introduced the idea of combining fuzzy and rough sets to develop soft similarity classes i.e., fuzzifying the approximations of rough set theory. Formally, a fuzzy rough set is a pair (*A*_1_, *A*_2_)∈(*X, R*) where A is a fuzzy set in *X* such that *R↓A* = *A*_1_ and *R↑A* = *A*_2_ and *R* is a fuzzy relation in *X* Cornelis et al. (2008). Fuzzy rough sets permit partial membership of an object to the lower and upper approximations and the approximate nature of information are modeled by means of fuzzy indiscernibility relations. In general, *R* can be considered as a fuzzy tolerance relation such *R*(*x, x*) = 1 and *R*(*x, y*) = *R*(*y, x*) for all x, y in *X*. Let *U* be the universe and *R* the fuzzy tolerance relation in *U* which is a mapping *U* → [0, 1] and A is a fuzzy set in *U*, the upper (*R↑A*) and lower approximation of A (*R↓A*) is calculated by *R* using different methods. The general form for this calculation from Jensen and Cornelis (2011) is as follows:


(1) $$\begin{array}{l} \left(R↓A\right)\left(x\right)=\underset{y\in U}{\inf}I\left(R\left(x,y\right),A\left(y\right)\right) \end{array}$$

(2) $$\begin{array}{l} \left(R↑A\right)\left(x\right)=\underset{y\in U}{\sup}T\left(R\left(x,y\right),A\left(y\right)\right) \end{array}$$

where I is an implicator and T is a t-norm which are fuzzy logic connectives crucial for fuzzy rough hybridization. The Kleen-Diennes Implicator implemented in the WEKA platform^1^ is defined as


(3) $$\begin{array}{l} T_{M}=min\left(x,y\right) \end{array}$$

(4) $$\begin{array}{l} I_{M}=max\left(1-x,y\right) \end{array}$$

In the Fuzzy Rough Nearest Neighbor (FRNN) implementation, given a set of conditional attributes *C*, R is defined as where *R*_*a*_ is the degree to which objects *x* and *y* are similar for attribute *a* Jensen and Cornelis (2011):


(5) $$\begin{array}{l} R\left(x,y\right)=\underset{a\in C}{\min}R_{a}\left(x,y\right) \end{array}$$

The two options for *R*_*a*_ are:


(6) $$R_{a}^{1}(x,y)=\exp(-\frac{{\left(a(x)-a(y)\right)}^{2}}{2\sigma_{a}^{2}})$$

(7) $$\begin{array}{l} R_{a}^{2}\left(x,y\right)=1-\frac{\left||a\left(x\right)-a\left(y\right)|\right|}{\left|a_{max}-a_{min}\right|} \end{array}$$

where $\sigma_{a}^{2}$ is variance of attribute a, *a*_*max*_, and *a*_*min*_ are maximal and minimal values of attribute a. We have used option 2 given Equation (7). For the sake of completeness, we use the FRNN algorithm presented in Jensen and Cornelis (2011) implemented in WEKA in the Supplementary Section 1.

## 3. Materials

### 3.1. Data preparation

The original dataset includes 541 unique patients and 32,514 records (for single and multiple visits). In this paper, patients with at least two different dates of a medical appointment with one of the Health Centers were considered (referred to as multiple visit dataset). The ages for youngest and oldest patients were 6 and 108 years respectively (with a mean value of 58.61). Additionally, this multiple dataset included 390 types of diagnoses with 75 unique cannabidiol formulations. After data cleaning, diagnoses types that were not of interest in this study removed, the multiple visit dataset was reduced to 354 patients from 375 patients. The final dataset after preprocessing for experimentation was: 8,281 records (2,911 male and 5,730 female).

- Patient Id : Since this feature uniquely identifies a patient, due to privacy reasons, this feature value was anonymized by removing each patient's name, date of birth, and any information that might reveal the patient's identity.
- Age: This feature gives the age of the patient where the minimum value for age is 6 and the maximum value is 108.
- Clinical Assessment Tool (CAT): This feature indicates the type of the clinical measure assessment tool that was utilized to assess and score the patient. Three specific CAT types were observed in this study; the GAD-7 (General Anxiety Disorder-7), PHQ-9 (Patient Health Questionnaire-9), and PSQI (Pittsburgh Sleep Quality Index).
- CAT Value : The feature gives the values for each of the CAT types: GAD-7, PHQ-9, and PSQI.
- CAT Observation Date: This feature gives the date on which a CAT value was observed.
- Sex Id: This feature gives the gender and the distribution of the patients coded as 1: male (34.2%) and 2: female (65.8%).
- Cannabinoid recommendation: This feature indicates the specific cannabinoid recommendation. The medical cannabis products contain varying amounts of cannabidiol (CBD) and tetrahydrocannabinol (THC), two phytocannabinoids found in cannabis.
- Diagnosis: This feature indicates the diagnosis of the patient. There were 390 types of diagnoses and only 13 types were considered in this research.

The raw data had several problems such as missing or invalid values, continuous values for dosage and similar diagnosis which required extensive preprocessing. In the following section, we discuss the preprocessing steps applied to the dataset.

### 3.2. Preprocessing

The description of the steps are as follows:

- Invalid and missing values: Invalid and null values were found in gender and CAT value features and were removed. For example, there were 114 records that gender had a value other than 1 or 2. Also, in the original dataset, there were 18 records that CAT value greater than 27. There were very few records with missing values which were also removed.
- Diagnosis coding: The raw data consisted of 390 diagnoses categories. Some low occurring or categories not relevant to this study were removed (e.g.: ADHD, MS, Anemia, Vitiligo, Blood Clot, Schizophrenia, and Overweight). Other granular categories such as migraine, classical migraine, common migraine, and chronic migraine without aura were combined into the broader migraine category. In this study, we were primarily interested in chronic pain, so patients with migraine and headache were included in the chronic pain category.
- Cannabinoid recommendation coding: The values for this feature were continuous since they represent dosage values. Since we were only interested in a broad class of values, these values were converted into integers using regular expressions (using Python regular expression package).
- Multiple cannabinoid recommendations: Many patients (almost 40%) were recommended more than one cannabinoid product for one particular diagnosis in a single visit. This was primarily for cannabinoid product classes CBD and CBD AND THC:CBD. For such patients, the recommendation was changed to CBD AND THC:CBD (category 3). This resulted in duplicate records and these duplicate records were removed.
- Multiple CAT values: Some patients had a different value for GAD-7/PHQ-9/PSQI during a single visit. For this feature, records with largest CAT value (most severe) were recorded.
- Time of visit: All time values with a small difference during a single visit were standardized and 21 patients had a slight time difference in at least one record.
- CAT value coding: This generated feature was designed to merge CAT value and CAT types: A0-A3, D0-D4, and S0-S3 to represent anxiety (GAD-7), depression (PHQ-9), and sleep disorder (PSQI) severity level respectively.

**Figure 1:**
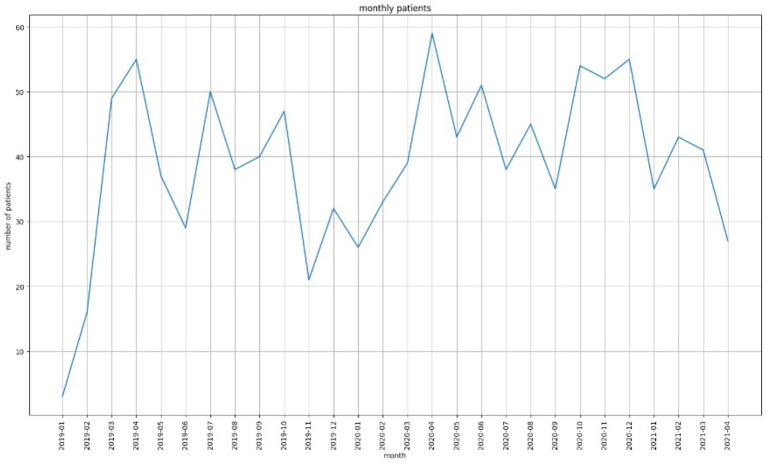
The number of patients that visited EKOSI Health Centers between January 2019 and March 2021. The highest number of visits recorded was during April 2020.

One of the main objectives of this study was to detect patterns in the fluctuations of values for GAD-7, PHQ-9 and PSQI (clinical assessment tools) for a patient during a time period. Figure 1 shows the number of patients from 2019 to 2020. As the figure shows, the number of patients that visited the Center was the highest (59) during March 2020 which was also the start of the first wave of COVID. In particular, we were interested in the overall outcome of a patient's quality of life in terms of whether their GAD-7/PHQ-9/PSQI scores were increasing/decreasing/constant during the period of observation. In addition, this information had to be co-related with their cannabinoid product recommendation and diagnosis.

**Figure 2:**
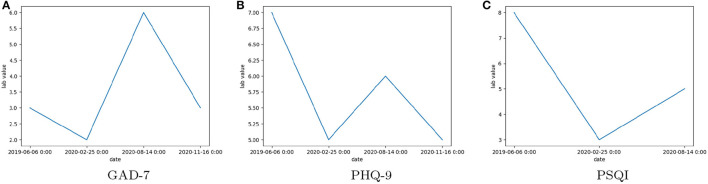
The observed scores of Clinical Assessment Tool Values: **(A)** General Anxiety Disorder 7 (GAD-7), **(B)** Patient Health Questionnaire 9 (PHQ-9), **(C)** Pittsburgh Sleep Quality Index (PSQI), for a single patient over multiple visits between June 2019 and November 2020.

Figure 2 shows the trends in score values for a single patient at the peak of COVID. It can be seen that in Figures 2A, B there is no regular pattern for GAD-7/PHQ-9/PSQI scores.

### 3.3. Engineered feature—patient status

To determine the outcome of a patient's treatment, we introduced a new feature (status) indicative of their progress or lack thereof due to the treatment received over a period of time. Three values for status were decided: Worse, Better, or No Change. An additional reason for introducing these labels was to train classification models so that these models can be used to determine (or predict) the status of a new patient. The flowchart for computation of the value of this this feature is given in Supplementary Section 2.2. The assumption behind this computation was that, since the score values for a disorder type does not follow any trend (as shown in Figure 2), a mean score value would be representative of a patient's score over the entire time period. In addition, there were unequal scores recorded for each patient during a time period. This problem was also observed for different disorder types as well. Hence, we separated the data into different CAT types first and then performed the labeling. This method also solved the problem of lack of observations of a CAT type with a time period for any given patient. The distribution of patient records based on i) labeled patient's status (Worse, Better, and No Change), ii) diagnosis (depression), and iii) CAT type for the four different types of cannabinoid formulations: CBD, THC:CBD, THC and CBD AND THC:CBD. The distribution of patients for other diagnoses (ex: Sleep Disorder, Chronic Pain, Arthritis, Anxiety, Depression) can be found in the Supplementary Section 3 and Supplementary Figures 5–9. However, chronic pain is the most frequent diagnosis and there were no patients with sleep disorder diagnosis who were recommended THC formulation.

## 4. Results

Results of the following algorithms are reported in Table 1: Random Forest, JRIP- Ripper algorithm in WEKA 3.7.2^2^ as well the classical rough sets model Rough Sets implemented in the Rough Set Exploration System (RSES 2.2.2).^3^ Many of the algorithmic methods used by RSES have their origins from rough set theory. The RSES software and underlying computational methods have been successfully applied in many studies and applications (Bazan and Szczuka, 2005). The most established classifiers in RSES are based on a set of decision rules. The LEM2 (Learning from Examples) rule-based algorithm is a covering technique that uses upper and lower approximation operators from rough sets to generate the classification results (Grzymała-Busse, 1992). The JRIP algorithm is an efficient implementation of the rule-based RIPPER (Repeated Incremental Pruning to Produce Error Reduction) algorithm (Cohen, 1995). Results from other fuzzy and rough sets algorithms implemented in WEKA are reported in the Supplementary Section 6. In Table 1, we provide the results (average values) in terms of classification accuracy (%), sensitivity(%), and specificity (%) for the four classifiers.

**Table 1:** Results—binary and ternary class experiments.

**Metric**	**Fuzzy rough NN**	**Random Forest**	**JRIP**	**Rough sets (RSES)**
Mean accuracy overall (binary)	97.11	96.22	97.16	99.20
Mean accuracy overall (ternary)	96.79	95.82	96.52	99.34
Accuracy (binary–better)	97.9	97.6	99.1	99.4
Accuracy (binary–worse)	95.7	93.9	93.8	98.78
Accuracy (ternary–better)	97.0	96.3	96.6	99.3
Accuracy (ternary–worse)	97.3	96.7	97.6	99.4
Accuracy (ternary–no change)	99.2	98.5	98.7	99.8
Sensitivity (binary–better)	97.9	97.6	99.1	99.4
Sensitivity (binary–worse)	95.7	93.9	93.8	98.78
Specificity (binary–better)	95.7	93.9	93.8	98.78
Specificity (binary–worse)	97.9	97.6	99.1	99.4
Sensitivity (ternary–better)	97.9	98.1	99.1	99.6
Sensitivity (ternary–worse)	95.0	93.6	93.5	98.7
Sensitivity (ternary–no change)	94.2	87.7	86.5	99.3
Specificity (ternary–better)	95.5	93.6	92.4	98.9
Specificity (ternary–worse)	98.2	98.0	99.4	99.7
Specificity (ternary–no change)	99.6	99.5	99.7	99.9

For the Random Forest classifier, the following parameters were used: maximum depth was set to 6 and number of trees was set to 10. For the JRIP classifier, one fold was used for pruning and two folds for growing the rules. For the FRNN classifier, 10 nearest neighbors were chosen, with Kleen-Diennes Implicator and Kleen-Diennes t-norm. For the results reported in Table 1, ten sets of training and testing pairs were used for experimentation across both platforms (10-Fold Cross Validation (CV) stratified method). We considered two forms of outcome of a patient's treatment: 2-class (Better or Worse) and 3-class (Worse, Better, or No Change) referred to as binary and ternary respectively. For the 2-class experiment, the Better class contains 5,157 records and the Worse class contains 3,124 records. For the 3-class, the Better class contains 5,157 records, Worse class contains 2,470 records and the No Change class contains 654 records.

- Average number of rules

- Binary classification: JRIP: 157, LEM2: 2,843
- Ternary classification: JRIP: 208, LEM2: 2,758.

- Execution time in secs per fold

- Fuzzy rough NN: 0.01, Random Forest: 0.19–0.25
- JRIP: 1-1.5, LEM2: 12.

## 5. Discussion

From the results in Table 1, the Rough Sets classifier (with LEM2 algorithm) gives the best overall result in terms of overall classification accuracy, accuracy per class, sensitivity, and specificity values for both the 2-class and the 3-class experiments. The best accuracy (99.34%) was obtained in the ternary classification. It is important to note in both cases (binary and ternary), the classification accuracy is over 99%. The class distribution in both experiments are highly imbalanced with the Better class having almost 2.5 times more records than the Worse class and 7.9 times the No Change class. The per class accuracy results are also consistently better in the ternary classification across all three classes. FRNN and JRIP classifiers are second best in terms of overall accuracy. The parameter settings for FRNN (number of K neighbors) and for JRIP were tuned to get the best results. Overall, FRNN gives the next best results. In terms of sensitivity (or the true positive rate), the best result for the ternary classification is with the better class (Rough Sets classifier- 99.6%). This is not surprising since there are more training examples for this class. In terms of specificity, the best result for the ternary classification is with the No Change class (or the true negative rate) is 99.9% which is consistent with the accuracy results for this class. Overall, rule-based models (RSES and JRIP) seem to do better that tree-based ensemble (Random Forest) and Fuzzy Rough Nearest Neighbor (FRNN) models for this dataset. However, the number of rules using the LEM2 algorithm is almost 13 times more than the JRIP classifier. This is also reflected in the time it takes for classification. In terms of execution time, FRNN is the best performing classifier. We have presented classification results of other nearest neighbor implementations using different forms of fuzzy and rough sets (Supplementary Table 4).

## 6. Conclusion

One of the objectives of this research was to predict whether new patients could potentially experience either an increase or decrease in clinical assessment tool scores. We demonstrate this by presenting results for 15 cases (ternary classification) with the LEM2 classifier in Supplementary Figure 8. Based on the results of a paired *t-*test (Supplementary Section 6), there is no statistical difference between FRNN and Rough Set classifier (highlighted in blue) in the binary case. For the ternary classification, there is a difference between Rough Set classifier and the other classifiers (JRIP, RF, and FRNN).

The combination of THC and CBD appears to be most beneficial on GAD-7, PHQ-9, and PSQI scores for patients dealing with anxiety, depression, sleep disorders, chronic pain, and arthritis. CBD alone overall had a positive effect on GAD-7, PHQ-9, and PSQI scores across all conditions but not as pronounced of a positive effect as a THC:CBD combination. THC alone worsened GAD-7 scores for all conditions except for arthritis patients which suggests THC may increase anxiety in patients. Other research has suggested that the combination of cannabinoids and terpenes or the “Entourage Effect” enhances the therapeutic potential of cannabinoids (Russo, 2019; Ferber et al., 2020). Our results appear to support this finding. Practically, this study highlights the need for additional research to further identify predictability, patterns and understand the efficacy and real-world evidence regarding cannabinoids, especially the combination of cannabinoids, for anxiety, depression, sleep disorders, chronic pain, and arthritis. We have identified rough-set based machine learning model with high accuracy that could be utilized for future studies regarding cannabinoids and precision medicine. Further research on the interaction and synergy of cannabinoids and terpenes, using this model, may lead to new and valuable insights, for the benefit of patients and health care practitioners alike.

The introduction of precise milligram cannabinoid dosing feature (numeric values), would lead to the application of discretization and normalization methods. Currently all patient features are nominal. The methodology to determine the engineered feature (status) for each patient would need further examination as the number of multiple visits for a patient would increase and fluctuate over time. Even though FRNN algorithm seems to be the best performing in terms of run-time performance, we believe that the rule-based algorithm (e.g., LEM2) facilitates easier interpretability of decisions in a clinical setting.

## Data availability statement

The raw data supporting the conclusions of this article will be made available by the authors, without undue reservation.

## Ethics statement

Approval for the secondary use of data was granted by the University of Winnipeg Human Ethics Research Board (UHREB) Protocol#WR001. Written informed consent from the participants' legal guardian/next of kin was not required to participate in this study in accordance with the national legislation and the institutional requirements.

## Author contributions

SR designed the materials and methods and writing of the paper. NA performed data processing and experiments. EL and KD provided and analyzed the data as well as assisted in editing the draft. ST contributed to the research. All authors contributed to the article and approved the submitted version.

## Footnotes

^1^ http://users.aber.ac.uk/rkj/book/wekafull.jar

^2^ https://www.cs.waikato.ac.nz/ml/weka/

^3^ https://www.roughsets.org/roughsets/software/
