# Supplementary material for: Rough-set based learning: Assessing patterns and predictability of anxiety, depression, and sleep scores associated with the use of cannabinoid-based medicine during COVID-19
Source: Front Artif Intell. 2023 Feb 15;6:981953. doi: 10.3389/frai.2023.981953 (PMC9975391; doi:10.3389/frai.2023.981953)
Supplement: Supplementary file 1 [file Data_Sheet_1.pdf]

Appendix. Rough-set based learning: Assessing patterns and predictability of anxiety, depression, and sleep scores associated with the use of Cannabinoid-Based Medicine during COVID-19

Sheela Ramanna [1], Negin Ashrafi [1,*], Evan Loster [2], Karen Debroni [2] and Shelley Turner [2]

[1]Department of Applied Computer Science, University of Winnipeg, WPG, MB, Canada
[2]Ekosi Health Centre Corporation, Winnipeg, WPG, MB, Canada

Correspondence*:
Sheela Ramanna
s.ramanna@uwinnipeg.ca

1 ROUGH SETS: UPPER AND LOWER APPROXIMATIONS

Figure 1 shows the regions that emerge with rough set approximation.

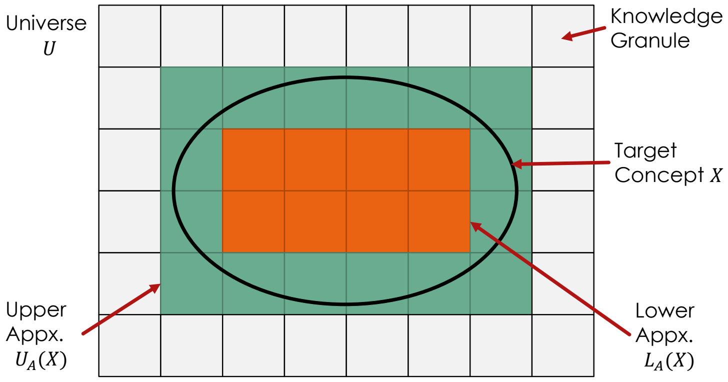

Figure 1. Rough Sets: Upper and lower approximations Ramanna et al. (2017)

Table 1 gives a list of symbols and their interpretation used in section 2.1 of the manuscript. Table 2 gives a list of symbols used in section 2.2 of the manuscript. Table 3 gives a sample patient data with three features (CAT type and value are combined) and one decision feature for illustration.

Table 1. Symbols - Rough Sets

Symbol	Interpretation
U	Finite, non-empty universe of objects (patients)
$R \subseteq U \times U$	Binary relation on the universe U
$\mathcal{A}$	Approximation Space
$X \subseteq U$	Target concept (patient status)
$[x]_R$	Equivalence class
$\mathcal{L}_{\mathcal{A}}(X)$	Lower approximation
$\mathcal{U}_{\mathcal{A}}(X)$	Upper approximation
$(\mathcal{U}_{\mathcal{A}}(X), \mathcal{L}_{\mathcal{A}}(X))$	Rough Set

Table 2. Symbols - Fuzzy Rough Sets

Symbol	Interpretation
U	Finite, non-empty universe of objects (patients)
$X \subseteq U$	Target concept (patient status)
R	Fuzzy tolerance relation in U where $U \rightarrow [0,1]$
A	Fuzzy Set in U
$(R \downarrow A)$	Lower Approximation
$(R \uparrow A)$	Upper Approximation
$((R \uparrow A), (R \downarrow A))$	Fuzzy Rough Set
$\mathcal{I}$	Implicator
$\mathcal{T}$	T-norm
inf	Infimum
sup	Supremum

Table 3. Sample Patient Data Table

Sample	CAT type/value	Diagnosis	cannabinoids recommendation	Status (decision)
x1	D2 (PHQ-9, 10-14)	Hypertension	CBD & THC: CBD	Better
x2	A3 (GAD-7, 15-21)	Chronic Pain	CBD	Worse
x3	S2 (PSQI, 10-15)	Fatigue	CBD & THC: CBD	Better
x4	D3 (PHQ-9, 15-19)	Sleep Disorder	CBD & THC: CBD	Worse
x5	A0 (GAD-7, 0-4)	Sleep Disorder	CBD & THC: CBD	Worse
x6	D3 (PHQ-9, 15-19)	Sleep Disorder	CBD & THC: CBD	Better
x7	A3 (GAD-7, 15-21)	Chronic Pain	CBD	Better
x8	D2 (PHQ-9, 10-14)	Anxiety	THC: CBD	Better
x9	A3 (GAD-7, 15-21)	Depression	CBD & THC: CBD	Worse
x10	D0 (PHQ-9, 0-4)	Hypertension	CBD & THC: CBD	Better

We illustrate the lower and upper approximations in terms of sample patient data presented in Table 3.

Example 1. Let $X = \{x1, x3, x6, x7, x8, x10\}$ for decision Better.
Let $[x]_R = \{x1, x2, x7, x3, x4, x6, x5, x8, x9, x10\}$ and

$\mathcal{A}$ = {CAT type-value, Diagnosis, cannabinoids recommendation}

$$\mathcal{L}_{\mathcal{A}}(X) = \{x1, x3, x8, x10\}.$$

$$\mathcal{U}_{\mathcal{A}}(X) = \{x1, x3, x4, x6, x2, x7, x8, x10\}.$$

$$\mathcal{B}_{\mathcal{A}}(X) = \{x5, x9\}.$$

The FRNN algorithm combines fuzzy rough approximations and fuzzy nearest neighbour algorithm which forms the basis for classification learning given below:

Algorithm 1: Fuzzy Rough Nearest Neighbor Classification (Jensen and Cornelis, 2011a)

Input : A decision table $\mathcal{A} = (U, A \cup d)$ as training data; y is new data
Output :Class is a predicted class
:

N←NN (y, K); /*NN is the k-nearest neighbor algorithm */
$\mathcal{T}$ ←0; Class← $\emptyset$; /*$\mathcal{T}$ from Eqn.7*/
foreach $C \in d$ do
  if $((R \downarrow C)(y) + (R \uparrow C)(y))/2 \geq \mathcal{T}$ then
    Class ← C;
    $\mathcal{T} \leftarrow ((R \downarrow C)(y) + (R \uparrow C)(y))/2$;
  end
  Output: Class

2 MATERIALS - ADDITIONAL DETAILS

2.1 Data Preparation

The following is the complete description of the features used in this study.

- Patient Id : Since this feature uniquely identifies a patient, due to privacy reasons, this feature value was anonymised by removing each patient's name, date of birth and any information that might reveal the patient's identity.
- Age: This feature gives the age of the patient where the minimum value for age is 6 and the maximum value is 108 and the mean value is 58. The dataset included 77 different age values. 68, 58, 77, 59, 39 with respectively 1677, 1281, 1275, 1231, 1110 records respectively.
- Clinical Assessment Tool (CAT): This feature indicates the type of the clinical measure assessment tool that was utilized to assess and score the patient. Three specific CAT types were observed in this study; the GAD-7 (General Anxiety Disorder – 7), PHQ-9 (Patient Health Questionnaire – 9), and PSQI (Pittsburgh Sleep Quality Index). The GAD-7 is a valid and efficient tool for screening for GAD and assessing its severity in clinical practice and research. The PHQ-9 is a reliable and valid measure of depression severity. This characteristic, plus its brevity, make the PHQ-9 a useful clinical and research tool. The PSQI is a self-rated questionnaire which assesses sleep quality and disturbances over a 1-month time interval. The

scores/severity levels for each of the CAT types and their diagnostic status are provided in Table 4.

- CAT Value : The feature gives the values for each of the CAT types: GAD-7, PHQ-9, and PSQI.
- CAT Observation Date: This feature gives the date on which a CAT value was observed.
- Sex Id: This feature gives the gender and the distribution of the patients coded as 1: male (34.2%) and 2: female (65.8%).There were 10,992 records were related to male patients and 19,230 records were related to female patients.
- cannabinoids recommendation: This feature indicates the specific cannabinoids recommendation. There were different formulations and dosages of cannabinoids products that were recommended. The medical cannabis products contained varying amounts of cannabidiol (CBD) and tetrahydrocannabinol (THC), two phytocannabinoids found in cannabinoids. Examples of formulations are CBD capsule, CBD oil, THC:CBD oil, yellow oil, red soft-gel THC, spray THC, CBD and 1:1 formulations. There were 75 types of prescribed medical cannabis products in the cannabinoids recommendations.
- Diagnosis: This feature indicates the diagnosis of the patient. There were 390 types of diagnoses. Only 13 types of diagnoses were considered in this study where some smaller categories, for example, high blood pressure and benign essential hypertension were combined into a broader group of hypertension. The diagnoses types were coded as follows: 0:Anxiety, 1:Depression, 2:Sleep Disorder, 3:Chronic Pain, 4:Arthritis, 5:Diabetes, 6:Gastro, 7:PTSD, 8:Hypertension, 9:Fatigue, 10:Epilepsy, 11:Autism, 12:Substance Dependence.

Table 4 provides scores/severity levels for each of the Clinical Assessment Tools and their diagnostic status that was used for our experiments.

Table 4. Scores/severity levels for each CAT Value and their diagnostic status

CAT Type	CAT Value Range(score)	Diagnostic Status
GAD-7	0 - 4	Minimal Anxiety
	5 - 9	Mild Anxiety
	10 - 14	Moderate Anxiety
	15 - 21	Severe Anxiety
PHQ-9	0 - 4	Minimal Depression
	5 - 9	Mild Depression
	10 - 14	Moderate Depression
	15 - 19	Moderately Severe Depression
	20 - 27	Severe Depression
PSQI	0 - 4	Good Sleep quality
	5 - 9	Poor Sleep Quality
	10 - 15	Bad Sleep Quality
	16 - 20	Severely Bad Sleep Quality
	21	Terrible Sleep Quality

2.2 Preprocessing Results

Figure 2 provides the flowchart for computing the value of Patient Status feature.

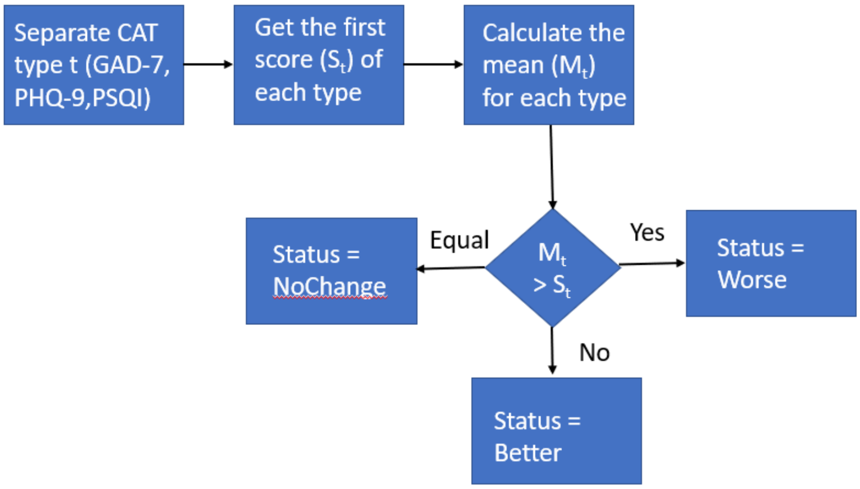

Figure 2. FlowChart - Patient Status Computation

The distribution of diagnosis and cannabinoids product recommendation classes at the beginning (before changing cannabinoids product recommendation and removing duplicates) are given in Figures 3, 4 respectively.

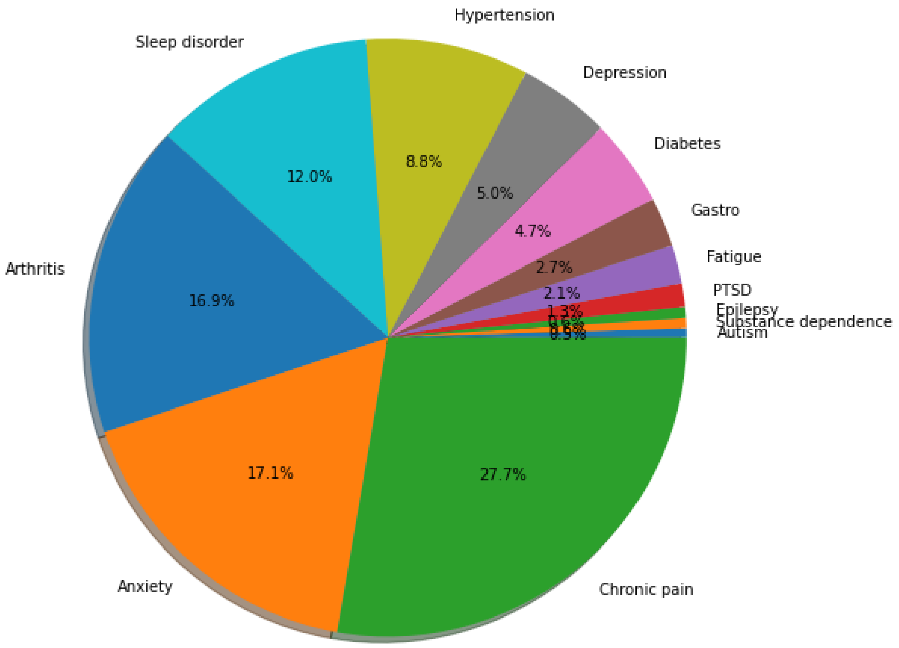

Figure 3. The distribution of patient diagnosis classes before data cleaning

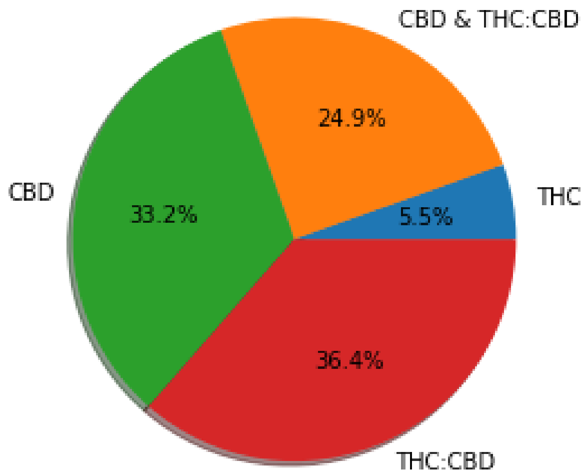

Figure 4. The distribution of patient cannabinoids product recommendation classes before data cleaning

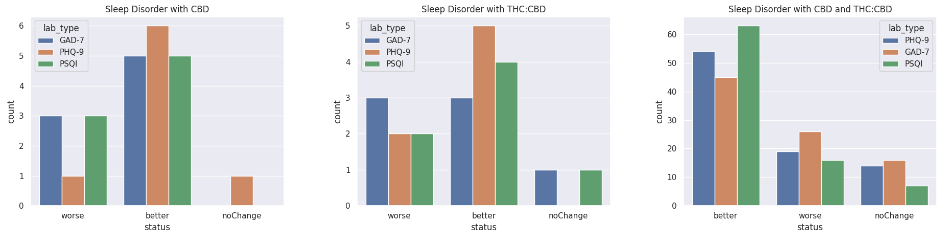

(5a) CBD (5b) THC:CBD (5c) CBD and THC:CBD

Figure 5. Distribution of patients with their status, diagnosed with Sleep Disorder

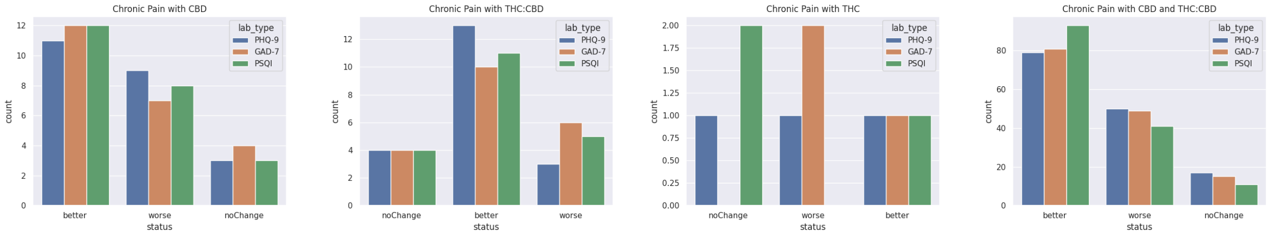

(6d) CBD (6e) THC:CBD (6f) THC (6g) THC:CBD

Figure 6. Distribution of patients with their status, diagnosed with Chronic Pain

3 DISTRIBUTION OF PATIENT RECORDS BASED ON DIAGNOSTIC STATUS

Figures 5, 6, 7, 8 and Figure 9 show the distribution of patient records based on i) labelled patient's status (worse, better, noChange) , ii) diagnosis, and iii) CAT type for the four different types of cannabinoids formulations: CBD, THC:CBD, THC and CBD AND THC:CBD . Also, note in Fig. 5, there were no patients with sleep disorder diagnosis who were recommended THC formulation.

[illegible]

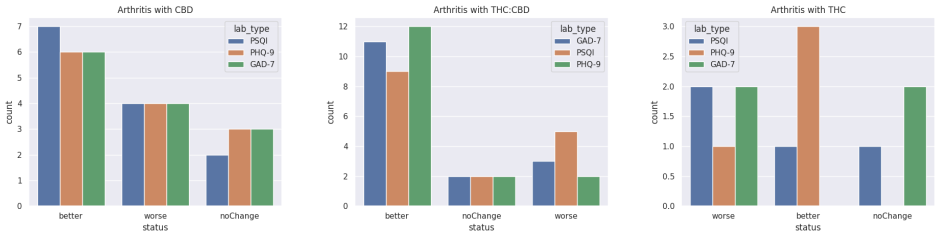

(7h) CBD (7i) THC:CBD (7j) THC (7k) THC:CBD

Figure 7. Distribution of patients with their status, diagnosed with Arthritis

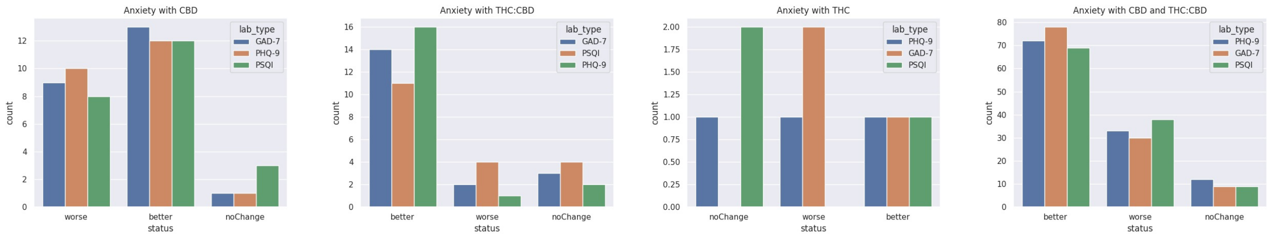

(8l) CBD (8m) THC:CBD (8n) THC (8o) CBD AND THC:CBD

Figure 8. Distribution of patients with their status, diagnosed with Anxiety

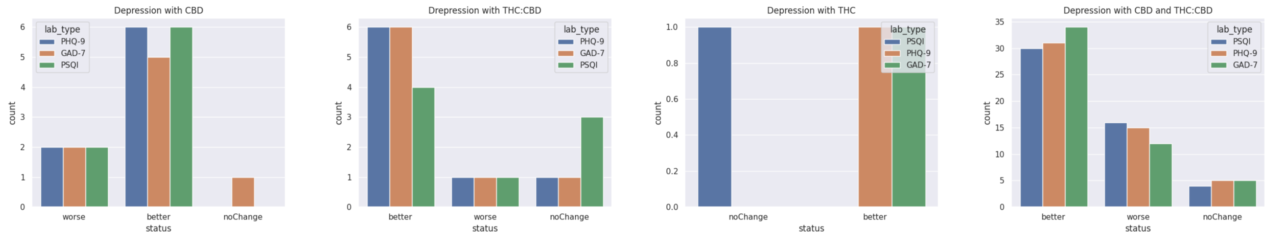

(9p) CBD (9q) THC:CBD (9r) THC (9s) CBD and THC:CBD

Figure 9. Distribution of patients with their status, diagnosed with Depression

4 OTHER CLASSIFIER RESULTS

Table 5 presents classification results of various nearest neighbour implementations using different forms of fuzzy and rough sets. where FURIA gives overall classification accuracy of over 95% which is again a rule-based classifier. The next best rule-based classifier is the discernibility classifier (DC).

Table 5. Results - WEKA FRNN implementations

Algorithm	Accuracy (3-class)	Accuracy (2-class)	Time 3 class (secs)	Time 2 class (secs)
FNN	78.13	81.17	0.01	0.03
FNN-O	89.51	90.69	0.01	0.01
VQNN	81.99	84.70	0.01	0.02
FURIA	95.17	96.60	60.8	61.4
DC	91.57	92.21	0.01	0.01

All results are based on 10-fold CV done automatically by the WEKA FRNN tool[1]. Here is a brief description of the methods.

- Vaguely Quantified Nearest Neighbour (VQNN) classifier (Jensen and Cornelis, 2011b) which is based on Vaguely Quantified rough sets (VQRS) algorithm introduced in (Cornelis et al., 2007). VQRS uses fuzzy linguistic quantifiers such as most and some to decide to what extent an object belongs to the lower and upper approximations.
- Fuzzy Unordered Rule Induction Algorithm (FURIA) FURIA extends the well-known RIPPER (Cohen, 1995) algorithm and learns fuzzy rules.
- Fuzzy nearest neighbour (FNN) algorithm was introduced to classify test examples based on their similarity to a given number K neighbours of training examples and membership degrees to (crisp or fuzzy) class labels of these neighbours (Keller et al., 1985).
- Discernibility Classifier (DC) - uses the discernibility matrix for deriving rules (Riza et al., 2014).

```
TABLE testset
ATTRIBUTES 9
date symbolic, lab type symbolic, lab type symbolic, lab val cat symbolic, sex id numeric 0,
age numeric 0, diagnosis numeric 0, CBD numeric 0, status symbolic
Objects 15
"2020-08-28 0:00" PSQI 8 2 53 8 3 better
"2020-12-16 0:00" PHQ-9 3 2 714 3 better
"2020-08-21 0:00" GAD-7 18 2 63 0 3 better
"2020-11-26 0:00" PHQ-9 11 2 63 2 3 better
"2020-08-25 0:00" PHQ-9 22 2 44 O 1 better
"2020-06-17 0:00" GAD-7 18 2 63 0 3 noChange
"2020-10-07 0:00" GAD-7 18 2 63 3 3 noChange
"2019-04-22 0:00" PHQ-9 2 2 55 O 3 noChange
"2019-09-24 0:00" GAD-7 0 1 68 2 3 noChange
"2019-09-16 0:00" GAD-7 0 2 60 4 3 noChange
"2019-07-07 0:00" PSQI 9 2 60 2 3 noChange
"2020-04-03 0:00" GAD-7 2 1654 3 worse
"2020-10-02 0:00" PSQI 14 1 42 0 3 worse
"2020-12-21 0:00" GAD-7 9 165 8 3 worse
"2020-09-10 0:00" PHQ-9 18 2 53 3 3 MISSING
```

Figure 10. Prediction results of unknown cases with Rough Sets

[1] http://users.aber.ac.uk/rkj/book/wekafull.jar

```
1   33  ("sex id"=2)&("lab type"=PSQI)&(cbd=1)&("lab val cat"=1S)=>(status={better[33]})
2   24  ("sex id"=2)&("lab type"=PHQ-9)&(cbd=1)&(diagnosis=0)=>(status={better[24]})
3   23  ("sex id"=1)&("lab type"=PHQ-9)&("lab val cat"=0.0)&(cbd=2)=>(status={better[23]})
4   20  ("sex id"=2)&("lab type"=GAD-7)&(cbd=3)&("lab val cat"=1A)&(age=51)=>(status={better[20]})
5   19  (cbd=3)&("sex id"=2)&("lab type"=PHQ-9)&("lab val cat"=3.0)&(age=51)=>(status={better[19]})
6   18  (cbd=3)&("sex id"=1)&("lab type"=PHQ-9)&("lab val cat"=0.0)&("lab value"=3)&(age=59)=>(status={better[18]})
7   17  ("sex id"=1)&("lab type"=PHQ-9)&("lab val cat"=0.0)&(cbd=1)=>(status={better[17]})
8   16  (cbd=3)&("sex id"=1)&("lab type"=PHQ-9)&("lab val cat"=0.0)&("lab value"=2)&(age=69)=>(status={better[16]})
9   16  ("sex id"=1)&(cbd=3)&("lab type"=GAD-7)&("lab val cat"=3A)=>(status={better[16]})
10  16  ("sex id"=2)&("lab type"=PHQ-9)&("lab val cat"=0.0)&(cbd=1)=>(status={better[16]})
11  16  ("sex id"=1)&("lab type"=PHQ-9)&(cbd=1)=>(status={better[16]})
12  16  ("sex id"=1)&("lab type"=GAD-7)&("lab val cat"=0A)=>(status={better[16]})
13  16  ("sex id"=2)&("lab type"=GAD-7)&("lab val cat"=0A)&("lab value"=0)&(cbd=2)=>(status={worse[16]})
14  16  ("sex id"=1)&("lab type"=PSQI)&(cbd=1)=>(status={worse[16]})
15  15  (cbd=3)&("sex id"=2)&("lab type"=GAD-7)&("lab val cat"=1A)&("lab value"=6)&(diagnosis=0)=>(status={better[15]})
16  15  (cbd=3)&("sex id"=2)&("lab type"=GAD-7)&("lab val cat"=2A)&("lab value"=14)&(age=51)=>(status={better[15]})
17  15  ("sex id"=2)&(cbd=3)&("lab type"=PHQ-9)&("lab val cat"=4.0)&("lab value"=24)=>(status={better[15]})
18  15  ("sex id"=2)&(cbd=0)&("lab type"=PHQ-9)&("lab val cat"=0.0)=>(status={worse[15]})
19  14  ("sex id"=1)&(cbd=3)&("lab type"=PHQ-9)&("lab val cat"=3.0)&("lab value"=16)=>(status={better[14]})
20  14  ("sex id"=2)&("lab type"=PSQI)&("lab val cat"=2S)&(cbd=1)=>(status={better[14]})
21  14  ("sex id"=2)&("lab type"=PSQI)&(cbd=3)&("lab val cat"=3S)=>(status={better[14]})
22  14  (cbd=3)&("sex id"=2)&("lab type"=PHQ-9)&("lab val cat"=0.0)&("lab value"=1)&(age=68)=>(status={worse[14]})
23  14  ("sex id"=1)&(cbd=3)&("lab type"=PHQ-9)&("lab val cat"=2.0)=>(status={worse[14]})
24  14  ("sex id"=1)&("lab type"=PSQI)&(cbd=3)&("lab val cat"=3S)=>(status={worse[14]})
25  13  ("sex id"=2)&(cbd=3)&("lab type"=PHQ-9)&("lab val cat"=4.0)=>(status={better[13]})
26  13  ("sex id"=1)&("lab type"=GAD-7)&(cbd=1)=>(status={better[13]})
27  13  (cbd=3)&("sex id"=1)&("lab type"=GAD-7)&("lab val cat"=2A)=>(status={worse[13]})
28  13  ("sex id"=2)&(cbd=0)&("lab type"=PSQI)&("lab val cat"=1S)=>(status={worse[13]})
29  13  ("sex id"=1)&(cbd=0)&("lab type"=GAD-7)&("lab val cat"=0A)&("lab value"=2)=>(status={worse[13]})
30  12  (cbd=3)&("sex id"=2)&("lab type"=PSQI)&("lab val cat"=1S)&("lab value"=9)&(age=52)=>(status={better[12]})
```

Figure 11. Sample rules with RSES

5 PREDICTION RESULTS

Figure 10 gives the prediction of unknown cases with RSES (LEM2 classifier). There are 8 features and one class feature (suppressed). Those cases that have a class label (Better, Worse, NoChange) were correct when compared with the actual values. It also has a label named MISSING. This is because the LEM2 method has only training coverage of 99% and may not cover all cases. However, of the cases that were classified, all of them were correct. Here we tested with samples with the class value removed. The coverage parameter for LEM2 was set to 0.99 for training. We also gave a sample set of rules generated by LEM2 in Figure 11.

6 T-TEST RESULTS

Tables 6 and 7 give the paired t-test results in terms of accuracy for the four classifiers for the two sets of experiments. The following parameters were used: two-tailed, $\alpha = 0.05$ and $n-1 = 9$ relative to 10 different training-testing runs, where n is the degree of freedom, where α is the significance level and probability distribution (Pr) value = 2.262. Based on the results of a paired t-test, for the binary classification, there is no difference between FRNN and Rough Set classifier (highlighted in blue). There is also no difference between other classifiers. For the ternary classification, there is a difference between Rough Set classifier and the other classifiers (JRIP, RF and FRNN). The reason for the difference is mainly due to need for parameter tuning with other method.

Table 6. T-test results: Comparison of classification accuracies for Binary Classification

Pairs	Average difference	T-stat
Fuzzy Rough NN – Random Forest	0.894	0.002
Fuzzy Rough NN - JRIP	0.048	0.900
Fuzzy Rough NN- Rough Set	2.091	1.717
Random Forest - JRIP	0.942	0.002
Random Forest – Rough Set	2.985	6.174
JRIP – Rough Set	2.043	5.855

Table 7. T-test results: Comparison of classification accuracies for Ternary Classification

Pairs	Average difference	T-stat
Fuzzy Rough NN – Random Forest	0.977	0.000
Fuzzy Rough NN - JRIP	0.277	0.381
Fuzzy Rough NN- Rough Set	2.546	5.198
Random Forest - JRIP	0.700	0.086
Random Forest – Rough Set	3.523	6.996
JRIP – Rough Set	2.823	4.881

REFERENCES

Cohen, W. (1995). Fast effective rule induction. In Proceedings of the 12th International Conference on Machine Learning, eds. A. Prieditis and S. Russell (Massachusetts: Morgan Kaufmann), 115–123

Cornelis, C., De Cock, M., and Radzikowska, A. M. (2007). Vaguely quantified rough sets. In Rough Sets, Fuzzy Sets, Data Mining and Granular Computing, eds. A. An, J. Stefanowski, S. Ramanna, C. J. Butz, W. Pedrycz, and G. Wang (Berlin, Heidelberg: Springer Berlin Heidelberg), 87–94

Jensen, R. and Cornelis, C. (2011a). Fuzzy-rough nearest neighbour classification. Transactions on rough sets XIII , 56–72

Jensen, R. and Cornelis, C. (2011b). Fuzzy-rough nearest neighbour classification and prediction. Theoretical Computer Science 412, 5871–5884

Keller, J., Gray, M., and Givens, J. (1985). A fuzzy k-nearest neighbor algorithm. IEEE Trans. Systems Man Cybernet. 15, 580–585

Ramanna, S., Peters, J. F., and Sengoz, C. (2017). Application of tolerance rough sets in structured and unstructured text categorization: a survey. In Thriving Rough Sets (Springer). 119–138

Riza, L. S., Janusz, A., Bergmeir, C., Cornelis, C., Herrera, F., Slezak, D., et al. (2014). Implementing algorithms of rough set theory and fuzzy rough set theory in the r package "roughsets". Information Sciences 287, 68–89
